# Disturbed cervical proprioception affects perception of spatial orientation while in motion

**DOI:** 10.1007/s00221-017-4993-5

**Published:** 2017-06-17

**Authors:** Eva-Maj Malmström, Per-Anders Fransson, Terese Jaxmar Bruinen, Semir Facic, Fredrik Tjernström

**Affiliations:** 1 Department of Pain Rehabilitation, Skåne University Hospital, Lund, Sweden; 2 Department of Otorhinolaryngology, Clinical Sciences, Lund University, Lund, Sweden; 3 Abels Rehab, Claesgatan 7, Malmö, Sweden; 4 Medpro Clinic Rehab AB, Torpavägen 23, Vänersborg, Sweden

**Keywords:** Orientation, Position sense, Spatial perception, Proprioception

## Abstract

The proprioceptive, visual and vestibular sensory systems interact to maintain dynamic stability during movement. The relative importance and interplay between these sensory systems is still not fully understood. Increased knowledge about spatial perception and postural orientation would provide better understanding of balance disorders, and their rehabilitation. Displacement of the body in space was recorded in 16 healthy subjects performing a sequence of stepping-in-place tests without any visual or auditory cues. Spatial displacement and orientation in space were determined by calculating two parameters, “Moved distance (sagittal + lateral displacement)” and “Rotation”. During the stepping-in-place tests vibration were applied in a randomized order on four different cervical muscles, and the effects were compared between muscles and to a non-vibration baseline condition. During the tests a forward displacement (“Moved distance”) was found to be the normal behavior, with various degrees of longitudinal rotation (“Rotation”). The moved distance was significantly larger when the vibration was applied on the dorsal muscles (916 mm) relative to on ventral muscles (715 mm) (*p* = 0.003) and the rate of displacement was significantly larger for dorsal muscles (36.5 mm/s) relative to ventral (28.7 mm/s) vs (*p* = 0.002). When vibration was applied on the left-sided muscles, 16° rotation to the right was induced (*p* = 0.005), whereas no significant rotation direction was induced with right-sided vibration (3°). The rate of rotation was significantly larger for vibration applied on ventral muscles (0.44°/s) relative to on dorsal (0.33°/s) (*p* = 0.019). The results highlight the influence of cervical proprioception on the internal spatial orientation, and subsequent for postural control.

## Introduction

Postural orientation and stability are dependent on complex interactions between the proprioceptive, visual and vestibular sensory systems to coordinate movements in response to varying demands and challenges (Cullen et al. 2011). Dysfunction in any of these systems cause suboptimal postural orientation, sometimes manifested as decreased stability or dizziness. Cervical proprioception is of special importance for spatial orientation, since it provides a reference frame, of how the head is positioned and moves relative to the trunk. This feature is essential for a correct interpretation of visual and vestibular inputs, e.g., for gaze stabilization during head movements (Mergner et al. 2001). Since vestibular afferents encode active and passive head movements identically, cervical proprioception acts as a reference to perceive the actual position of the head relative to the trunk (Mergner and Rosemeier 1998; Cullen et al. 2011). Proprioception is also important for performing coordinated, correctional and targeted intersegmental movements (Fortier and Basset 2012). The abundance of proprioceptors in the cervical region, in muscles and joints, reflects the special importance of detailed information from this region for fine-tuned movement control (McLain 1994; Kulkarni et al. 2001; Boyd-Clark et al. 2002). Disturbed proprioceptive information can induce illusions of distorted body perception (Lackner 1988). Pain of different etiology influences cervical proprioception (Revel et al. 1991; Heikkila and Astrom 1996; Rix and Bagust 2001; Lee et al. 2008; Paulus and Brumagne 2008; Malmstrom et al. 2013), sometimes manifested as dizziness due to sensory mismatch (Reason 1978) and denoted as “cervicogenic dizziness” (Karlberg et al. 1996; Wrisley et al. 2000; Brandt and Bronstein 2001; Reid and Rivett 2005; Malmstrom et al. 2007; Lystad et al. 2011). However, the lack of reliable clinical tests and the uncertainty about the impact of disturbed cervical proprioception on spatial orientation, still makes “cervicogenic dizziness” debatable (Brandt and Bronstein 2001; Yacovino and Hain 2013).

To evaluate the contribution of cervical proprioceptive contribution for spatial orientation, it is necessary to exclude/reduce other sensory information. Visual information is easily eliminated by closure of the eyes and by the concurrent use of a blindfold. Mechanoreceptive information can partly be reduced while performing a stepping-in-place test, since during stepping the feet only have a brief period of contact with the support surface. During experimental conditions cervical disturbances have been shown to modify gaze direction (Biguer et al. 1988), as well as body-centered coordination (Bove et al. 2002). The existence of a short-latency integrative system between cervical proprioception and the activation of postural muscles has been suggested for postural control (Magnusson et al. 2006), and cervical proprioception have been shown to modulate vestibular-dependent motion perception (Pettorossi et al. 2015).

The cervical proprioceptive capacity can indirectly be tested with sensorimotor tests, commonly by testing the ability to reproduce different predetermined head on trunk positions (Revel et al. 1991; Loudon et al. 1997; Dvir and Prushansky 2000; Malmstrom et al. 2013). The importance of cervical proprioception for postural orientation can be tested by different perturbations of cervical muscles (Wierzbicka et al. 1998; Strupp et al. 1999; Ivanenko et al. 2000; Bove et al. 2002; Karnath et al. 2002; Magnusson et al. 2006; Fransson et al. 2007; Patel et al. 2010). Perturbations with vibration applied on muscles have been shown to produce a response proportional to the vibration frequency and the vibration amplitude (Roll and Vedel 1982; Fransson et al. 2007). Vibration (at 70–100 Hz frequencies) stimulates the muscle spindle, considered the most important proprioceptor (Roll and Vedel 1982). Vibration over cervical muscles do not cause any recorded concomitant stimulation of the vestibular organs, which could have been suspected due to their close proximity (Magnusson et al. 2006) The effect of vibratory induced perturbations on cervical muscles during a stepping-in-place test could contribute to a deeper understanding of the plasticity of spatial orientation and how human postural control over time withstands proprioceptive disruptions.

The overall aim of the study was to investigate the influence of cervical proprioceptive perturbation on postural control and its importance for the perception of spatial orientation while moving. We wanted to examine the potential causal relationship between perturbed cervical proprioception and altered spatial orientation.

## Materials and methods

### Subjects

Sixteen young healthy subjects participated in the study (8 men, 8 women, mean age 24 (19–34 years); mean height 175 cm (160–194 cm); mean weight 73 kg (55–104 kg). They were recruited through advertisement or through personal recruitment at the university, at workplaces and at spare time activities. The subjects considered themselves as healthy and had no ongoing neck pain, nor a history of neck pain conditions or injuries to the head or neck, or any symptoms from the vestibular system such as dizziness or imbalance, or any injuries/sensory losses in the lower limbs. All subjects were right-handed. They got uniform information about test setup and procedures and were informed that they could stop the participation during the test at any time and for any reason, without giving any explanation. Informed consent was obtained from all individuals included in the study. The study conforms to the standards set by Declaration of Helsinki, 2004 and was approved by the ethics board at the Health Sciences, Lund, Sweden and Regional Ethics Review Board, Lund University, Lund, Sweden (Vårdvetenskapliga Etiknämnden, §LU189-00, §LU65-1989, 411/2006). All personal confidential data were handled according to the Personal Data Act (1998:204).

### Experimental design

The spatial position and rotation of the body around its own longitudinal axis was continuously recorded in real-time while performing a modified Fukuda stepping-in-place test (STIP) (Fukuda 1959; Bonanni and Newton 1998; Dvir and Prushansky 2000; Bove et al. 2002; Weber et al. 2002). The STIP was conducted either with or without vibration directed to the dorsal or ventral cervical muscles at either side of the neck in a randomized order.

**Fig. 1:**
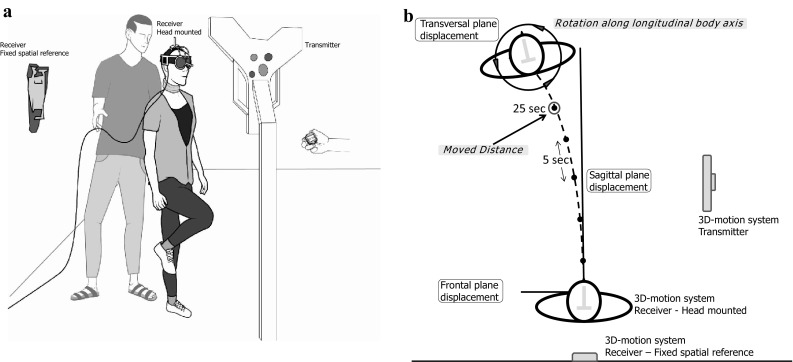
**a** The stepping-in-place tests (STIP) were performed with the subjects blindfolded and with vibrators applied on ventral and dorsal cervical muscles using a flexible neck attachment. **b** Subjects spatial drift during STIP, quantified as moved distance and as rotation along the longitudinal body axis at 5-s intervals (see *dots*). The moved distance was calculated from the 3D-motion system coordinates for sagittal and frontal displacement in mm and from the 3D-motion system recordings of longitudinal rotation in the transversal plane in degrees

The STIP was performed in a quiet room, without any distracting or external reference cues. Before the tests were performed, the subjects were allowed to familiarize themselves with the equipment and test procedures. The subjects got identical oral instructions (read out loud from a protocol by the examiner), about stepping in place with alternating leg lifts at similar pace and with foot lifts resembling the height of stair steps. Lesser steps than the original 100, introduced by Fukuda himself (Fukuda 1959) has been suggested (Bonanni and Newton 1998; Paquet et al. 2014). We have adopted the suggestions by Bonanni (Bonanni and Newton 1998), using 50 steps with the modification of average time for 50 steps (35 s). In a familiarization STIP the subjects performed 50 steps (Bonanni and Newton 1998), guided by the examiners counting, who also if necessary corrected the pace or height of the steps. Then the recording devices and vibrators were attached to the subjects and they were blindfolded and also asked to close their eyes. The subjects wore earplugs to minimize external audiological reference cues, but were still able to hear verbal instructions. The subjects were spoken to during the test only for safety reasons and then directly from the front and from the same distance throughout the test. The subjects used comfortable shoes and stood upright with feet together at the start of the tests, with their arms hanging down alongside the body (Fig. 1a).

Each stepping test was performed during 35 s. This time was the median time for a 50-stepping-in-place test in a pre-study, after recommendations by Bonanni (Bonanni and Newton 1998), to achieve comparable recordings in the Zebris^®^ system. Of those 35 s, the initial 25 s of the recordings were used in the final analysis (Fig. 1b). Twenty-five seconds were regarded best suitable as the endpoint of displacement quantification, since some of the subjects moved outside the measuring range or angle of the equipment during longer performance. After each stepping test the subjects were helped to sit down on a mobile office chair, resting their feet on a footrest. Then the chair was moved back to the initial starting position by the test leader, with random movements in order not to give any informational cues about the actual spatial displacement made during the previous STIP.

The subjects performed, in one uninterrupted sequence, the STIP 3 times (denoted I, II, III) for each of the five test conditions assessed (non-vibration; vibration ventral/dorsal, right/left). The non-vibration condition was always the initial, defined as baseline, followed by the vibration conditions, performed in randomized order (Latin square design: ventral/dorsal and right/left). To eliminate any residual vibration effect from previous tests, a “wash-out” 35 s STIP without vibration was performed before a new vibration condition (Rogers et al. 1985; Ribot-Ciscar et al. 1998).

### Disturbance of cervical proprioception by vibration

The vibrators were attached over the sternocleidomastoid muscle ventrally (denoted ventral muscles) and the paraspinal muscles at C2–3 level dorsally (denoted dorsal muscles) with adhesive tape (K-Active Sweden). No contact with the head nor the spine was allowed. The attachment was reinforced with an elastic pad (Tubigrip), securing the vibrators to stay in place during the test. The vibrators were attached alongside over the middle of the sternocleidomastoid muscle belly and attached horizontally across the unilateral paraspinal musculature at C2–C3 level. Cylindrical, high intensity vibrators (60 × 10 mm) producing a vibration of 1.0 mm amplitude and at a frequency of 85 Hz were used in the study. During all vibration conditions, the vibration was continuously on during the entire 35 s STIP, with the start and ending of the vibration in close connection to the STIP.

### Assessment of spatial body position and longitudinal rotation

The spatial body position in 3D space and rotation around the body‘s own longitudinal axis was recorded in real-time by a 3D motion analysis system, Zebris^®^ (Zebris^®^ CMS-HS, with software Win-Spine, version 1.78; Zebris Medizintechnik GmbH, Isny, Germany) (Dvir and Prushansky 2000). The recording equipment consisted of a helmet and a shoulder cap, equipped with three ultrasound microphones that made determination of the absolute position in space and body rotation in 3D space possible. The Zebris^®^ helmet was attached on the subject’s head (body position in space and rotation), and the Zebris^®^ shoulder cap was attached to the wall (reference), just behind the subjects in their starting position (Fig. 1). The microphones received signals from three transmitters on a frame, positioned approximately 1.5 m to the right of the subject. The sampling frequency of position and rotation data was 50 Hz. The Zebris^®^ measures distances to the microphones according to the principle of the timing of the intervals between the emission and the reception of ultrasound pulses. The absolute 3D coordinates are then calculated by triangulation. The 3D motion analysis system records the subject’s spatial position within a distance of 2.5 m from the transmitter in all 3 dimensions, with a resolution better than ±1 mm, and records the subject’s angular orientation with a resolution of about ±0.1 degrees in all 3 dimensions (Zebris^®^ CMS-HS manual; Zebris Medizintechnik GmbH, Isny, Germany). The movement trajectory during STIP was typically within 1.0–1.5 m from the transmitter stand and deviated never more than 1.8 m from the stand for any subject and test condition investigated.

Notably, during all tests, the subjects kept their head position still, relative to the trunk, and thus, the rotation values always represent body rotation as a whole around the body's longitudinal axis.

### Data analysis

Displacement emanated during the STIP from a body-centered perspective and consequently, was regarded as an expression for the perception of the actual body position in space.

The body position was quantified at 5-s time interval, i.e., at the starting point (0 s) and thereafter at five defined time points (5, 10, 15, 20, 25 s). The exact spatial displacement and the orientation of the body in space at each of these time points were determined by calculating two parameters, defined as “Moved distance” and “Rotation”. “Moved distance” represents the subject’s total displacement in absolute terms, calculated from the frontal and sagittal positions with reference to the start position at 0 s. It was summarized into one value for each time sample, using Pythagorean Theorem (Fig. 1b). “Rotation” represents orientation of the body along the longitudinal axis, assessed and presented by the Zebris^®^ software in degrees. Positive values represent a rotation towards the right (clockwise) with reference to the initial 0 degree position and negative values represent a rotation towards the left (counter-clockwise).

A statistical analysis of the three repetitions of STIP during each test condition (denoted I-III) revealed that repeating the STIP in sequence produced no significant order effect when subjected to vibration. Thus, to reduce measurement artifacts, the mean value for the three repeated STIP during the same test condition were calculated for the six defined time points (0, 5,…,25 s) for each of the test conditions, and used in the final analysis. When the first STIP was performed at baseline, STIP I differed considerably from STIP II and III, which forms the rationale to use data only from STIP II and III in analyses when calculating moved distance and rotation and when comparing with conditions with vibration.

### Statistics

The two parameters “Moved distance” and body “Rotation” were evaluated with multivariate analyses to determine the role of both main factors and main factor interactions using two-step procedures: (1) an initial repeated measures GLM ANOVA (General Linear Model Analysis of Variance) followed by (2) post hoc within-individuals evaluations. Twelve GLM ANOVA + post hoc analyses (six for each signed parameter “Moved distance” and body “Rotation”) were done to: (I) determine the applicable baseline performance without vibration; (II) determine the performance and differences between vibration applied at four different cervical muscle sites, (III) compare the performance when vibration was applied on each of the different cervical muscle sites to the recorded baseline performance without vibration.

For analysis (I), a GLM model including one main factor “Time”, (0, 5,…,25 s; *df* 5) evaluated if “Moved distance” and body “Rotation” systematically changed over time during STIP.

For analysis (II), a GLM model including three main factors “Muscle” (ventral, dorsal; *df* 1); “Side” (left, right; *df* 1); and “Time”, (0, 5,…,25 s; *df* 5) and their interactions evaluated if the kind of muscle submitted to vibration changed “Moved distance” and body “Rotation” and if the vibrated muscle site had a weaker or stronger effect over time.

For analysis (III), four GLM models including two main factors “Vibration” (no vibration; vibration, *df* 1); and “Time”, (0, 5,…,25 s; *df* 5) and their interactions evaluated if vibration submitted to any of the four muscle sites made the performance of “Moved distance” and the body “Rotation” change compared to the baseline performance without vibration and if so, if the vibrated muscle site had a weaker or stronger effect over time.

Moreover, as part of the post hoc evaluation, the best fitting dynamic patterns and time constants describing the changes in “Moved distance” and the body “Rotation” over time were determined using linear or exponential regression models. The regression analysis provides information about the robustness of spatial orientation over time. If the deviations are linear over time, then this suggest that there is a fairly constant bias in spatial perception, but also that there is a certain robustness in the performance. However, if the deviations are exponential, this may indicate that, e.g., the ability to sense rotation after a certain point in time may be dramatically reduced. Longitudinal rotation was in the population fairly equally distributed between right (positive values, clockwise) and left rotations (negative values, counter-clockwise). Thus, when analyzing the temporal properties of the body rotation we found reasons to analyze both signed and absolute rotation values. Additionally, the Wilcoxon signed-rank tests (Exact two-tailed) was used for within-individuals post hoc comparisons. Non-parametric statistical tests were used as some data sets were not normally distributed following the Shapiro–Wilk test.

In all GLM ANOVA tests, *p* < 0.05 was considered significant. In the Wilcoxon comparisons *p* < 0.05 was considered significant when evaluating effect of muscle and side location, whereas *p* < 0.0125 was considered significant when evaluating individual effects of vibration on muscle sites vs. baseline performance, following Bonferroni correction principles.

A sample size analysis using G-power™ evidenced a power value of 0.8 for both parameters “Moved distance” and “Rotation”.

## Results

### Moved distance and longitudinal rotations without vibration

A pre-analysis of the data revealed an initial order effect when determining the baseline performance. The performance during the first stepping sequence (I) differed from the second (II) and third (III) sequence, at which the performance were near identical between repeated tests. When analyzed, there was a significant effect of repetition on moved distance for STIP I, II, III (*p* = 0.001, *F* 15.5), but not for STIP II, III (*p* = 0.206, *F* 1.7). Thus, the applicable baseline performance without vibration was determined best reflected by averaging the data on sample level from STIP II and III (Table 1).

**Table 1:** Effects of muscle category (dorsal, ventral; left, right) and time on the parameters moved distance and longitudinal rotation

	Parameters		Statistical results: no vibration vs. muscle vibration					
			Moved distance^a^			Rotation^a^		
	Moved distance (mm)^b^	Rotation (°)^b,c^	Vibration	Time	Vibration × time	Vibration	Time	Vibration × time
No vibration (STIP I)	628 (63)	−13.6 (7.0)	–	–	–	–	–	–
No Vibration^d^ (STIP II, III)	804 (78)	−3.6 (5.8)	–	<0.001 (99.7)	–	–	0.531 (0.4)	–
Dorsal left	911 (82)	13.2 (5.4)	0.023 (6.4)	<0.001 (123.6)	0.006 (10.1)	0.001 (18.9)	0.201 (1.8)	0.002 (13.3)
Dorsal right	920 (93)	3.1 (5.9)	0.019 (6.9)	<0.001 (107.4)	0.004 (11.2)	0.274 (1.3)	0.091 (3.3)	0.005 (10.5)
Ventral left	693 (70)	17.8 (6.8)	0.105 (3.0)	<0.001 (113.7)	0.013 (7.9)	0.005 (10.5)	0.143 (2.4)	0.023 (6.5)
Ventral right	737 (74)	3.0 (7.2)	0.350 (0.9)	<0.001 (110.8)	0.065 (4.0)	0.259 (1.4)	0.360 (0.9)	0.097 (3.1)

^a^The analyses evaluated the effects of main factors: Vibration, Time and the interaction effect between main factors Vibration × Time [*p* values and (*F* values)]

^b^Moved distance and longitudinal rotation [mean and (SEM)]

^c^Positive values represent rotation towards the right (clockwise), negative values represent rotation towards the left (counter-clockwise)

^d^STIP II, III in the non-vibration condition = baseline

**Fig. 2:**
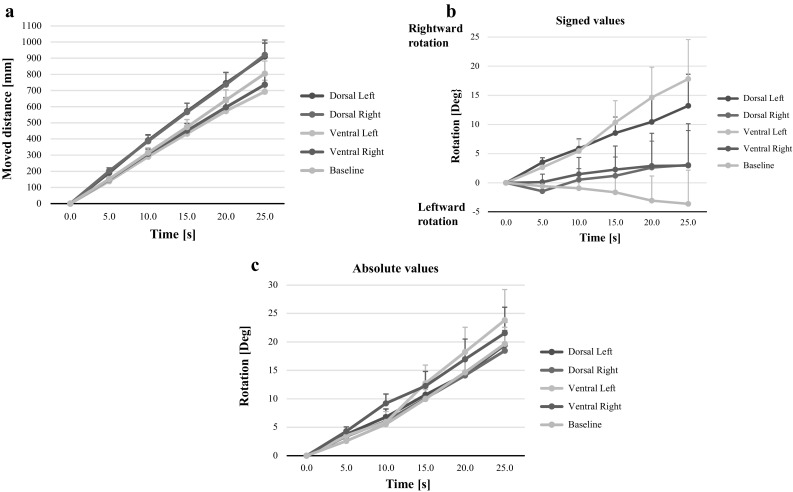
**a** Moved distance during each test condition (mean and SEM values). The moved distance increased during dorsal vibration relative to the baseline condition and diminished during ventral vibration relative to the baseline condition. **b** Signed longitudinal rotation (mean and SEM values). The signed values show that vibration on the left sided muscles was the only test condition that produced a systematic directional rotation while performing STIP. **c** Absolute longitudinal rotation (mean and SEM values). Vibration on ventral muscles caused a slightly larger rotation compared to vibration on dorsal muscles

The “Moved distance” increased significantly over “Time” (*p* < 0.001, *F* = 99.7) during the baseline recordings, resulting in a total displacement of 804.5 mm (SEM 78.3) during the 25 s assessed (Table 1; Fig. 2a). However, on group level “Rotation” was not significantly systematically changed over “Time” (*p* = 0.531, *F* = 0.4) from the initial ‘straight ahead orientation’ during baseline recordings, but individually rotations were seen in both directions with an average rotation during STIP by 3.6 degrees (SEM 5.8) to the left (Table 1; Fig. 2c).

### Moved distance and longitudinal rotations with vibration

#### Moved distance: repeated measures GLM ANOVA

The “Moved distance” was significantly larger (*p* = 0.003, “Muscles”) when the vibration was applied on the dorsal muscles relative to the ventral muscles (Table 2). Moreover, the “Moved distance” increased significantly over time (*p* < 0.001, “Time”) when vibration was applied.

**Table 2:** Effects of time and vibration applied on dorsal and ventral muscles, left and right side, on the parameters moved distance and longitudinal rotation

Parameters	Statistical results: vibration						
	Muscle	Side	Time	Muscle × Side	Muscle × Time	Side × Time	Muscle × Side × Time
Moved distance^a^	0.003 (12.5)	0.424 (0.7)	<0.001 (119.2)	0.388 (0.8)	0.002 (13.2)	0.025 (6.2)	0.173 (2.0)
Rotation^a^	0.597 (0.3)	0.005 (10.8)	0.061 (4.1)	0.713 (0.1)	0.019 (6.9)	0.012 (8.1)	0.009 (9.0)

^a^The analyses evaluated the effects of main factors: Muscle (dorsal, ventral), Side (left, right), Time, and all combinations of interactions between main factors [*p* values and (*F* values)]

The GLM ANOVA interactions revealed that the “Moved distance” increased significantly faster over time (*p* = 0.002, “Muscles × Time”) when the vibration was applied on the dorsal muscles relative to the ventral muscles. Furthermore, the “Moved distance” increased significantly faster over time by (*p* = 0.025, “Side × Time”) when the vibration was applied on the right muscles relative to the left muscles.

#### Moved distance: post hoc evaluations

The post hoc evaluation revealed that the “Moved distance” was significantly longer during STIP with dorsal vibration than during ventral vibration (*p* < 0.001). Moreover, vibration applied on the right side produced a significantly longer “Moved distance” than vibration applied on the left side (*p* = 0.014).

#### Rotation: repeated measures GLM ANOVA

The “Rotation” was significantly larger to the right (*p* = 0.005, “Side”) when the vibration was applied on the left side muscles relative to the right side muscles.

The GLM ANOVA interactions revealed that the “Rotation” increased significantly faster to the right over time (*p* = 0.019, “Muscles × Time”) when the vibration was applied on the ventral muscles relative to the dorsal muscles. Furthermore, the “Rotation” increased significantly faster to the right over time (*p* = 0.012, “Side × Time”) when the vibration was applied on the left muscles relative to the right muscles. Finally, the “Rotation” increased significantly faster to the right over time (*p* = 0.009, “Muscle × Side × Time”) when the vibration was applied on the left ventral muscle relative to the average values found when the vibration was applied on the other muscle sites.

#### Rotation: post hoc evaluations

The post hoc evaluation revealed that the “Rotation” was not significantly different between dorsal and ventral vibration (*p* = 0.651). However, vibration applied on the left side produced a significantly larger “Rotation” to the right than vibration applied on the muscles on the right side (*p* < 0.001).

### Moved distance and longitudinal rotations comparing with and without vibration

#### Moved distance: repeated measures GLM ANOVA

The “Moved distance” was significantly larger (*p* = 0.023, “Vibration”) when the vibration was applied on the dorsal left muscles relative to the baseline (Table 1). Similarly, the “Moved distance” was significantly larger (*p* = 0.019, “Vibration”) when the vibration was applied on the dorsal right muscles relative to the baseline.

The “Moved distance” increased significantly over time (*p* < 0.001, “Time”) for all vibration test conditions when analyzed together with the test baseline data in the GLM model (Fig. 2a).

The GLM ANOVA interactions revealed that the “Moved distance” increased significantly faster over time when vibration was applied on dorsal muscles relative to the baseline (*p* = 0.006, vibration left side; *p* = 0.004, vibration right side) (“Vibration × Time”). The “Moved distance” increased significantly slower over time when a vibration was applied on the ventral left muscles relative to the baseline (*p* = 0.013, vibration left side) (“Vibration × Time”) (Fig. 2a).

#### Moved distance: post hoc evaluations

The post hoc evaluation revealed that the “Moved distance” was significantly longer during STIP with dorsal left side vibration (*p* < 0.001) and dorsal right side vibration (*p* < 0.001) relative to the baseline condition. Moreover, the “Moved distance” was significantly shorter during STIP with ventral left side vibration (*p* = 0.005), but not during ventral right side vibration (*p* = 0.387) compared with the baseline test.

#### Rotation: repeated measures GLM ANOVA

The “Rotation” was significantly larger to the right (*p* < 0.001, “Vibration”) when the vibration was directed to the dorsal left muscles relative to the baseline. Similarly, the “Rotation” was significantly larger to the right (*p* = 0.005, “Vibration”) when the vibration was directed to the ventral left muscles relative to the baseline. The “Rotation” did not change significantly over time (*p* ≥ 0.091, “Time”) for any cervical vibration test conditions relative to the baseline data in the GLM model (Table 1).

The GLM ANOVA interactions revealed that “Rotation” increased significantly faster over time to the right when vibration was applied on the dorsal left muscles (*p* = 0.002, “Vibration × Time”) and to the ventral left muscles (*p* = 0.023, “Vibration × Time”) relative to the baseline. However, the “Rotation” was significantly slower over time to the right (*p* = 0.005, “Vibration × Time”) when vibration was applied on the dorsal right muscles relative to the baseline condition. Of note, without vibration the predominant rotation during STIP was to the left, although not significantly so (Table 1).

#### Rotation: post hoc evaluations

The post hoc evaluation revealed that the “Rotation” was significantly larger to the right during STIP with dorsal left side vibration (*p* < 0.001), but not during dorsal right side vibration (*p* = 0.143) compared to the baseline condition. Moreover, the “Rotation” was significantly larger to the right during STIP with ventral left side vibration (*p* < 0.001), but not during ventral right side vibration (*p* = 0.182) compared to baseline test.

### Resilience and dynamic component of spatial orientation

#### Moved distance

We found that a linear regression model best described the changes over time when analyzing the temporal properties of moved distance in detail (Table 3). The moved distance during the baseline (time constant 32.3 mm/s, *p* < 0.001) increased by dorsal vibration to 36.5 mm/s (right and left vibration, *p* < 0.001), and decreased by ventral left vibration to 28.0 mm/s (*p* < 0.001), and by ventral right vibration to 29.5 mm/s (*p* < 0.001).

**Table 3:** Regression analysis of changes in moved distance and longitudinal rotation over time

Regression model	Vibration site/state	*p* value^a^	Time constant (mm/s)
Linear			
Moved distance—signed values^b^	Dorsal left	<0.001 (236.6)	36.5
	Dorsal right	<0.001 (193.1)	36.5
	Ventral left	<0.001 (206.0)	28.0
	Ventral right	<0.001 (196.9)	29.5
	Baseline	<0.001 (214.5)	32.3

Linear			(º/s)
Rotation—signed values^c^	Dorsal left	0.002 (10.4)	0.51
	Dorsal right	0.779 (0.1)	0.16
	Ventral left	<0.001 (14.8)	0.74
	Ventral right	0.996 (0.0)	0.14
	Baseline	0.114 (2.5)	−0.15

Exponential			(º/s)
Rotation—absolute values	Dorsal left	<0.001 (62.9)	0.036
	Dorsal right	<0.001 (70.8)	0.036
	Ventral left	<0.001 (70.6)	0.043
	Ventral right	<0.001 (70.0)	0.039
	Baseline	<0.001 (147.3)	0.041

^a^ *p* values and (*F* values) are reported

^b^Data sets with “Moved distance” included no negative values, thus, regression analyses were made only on signed values

^c^Positive values represent longitudinal rotation towards the right (clockwise), negative values represent rotation towards the left (counter-clockwise)

#### Longitudinal rotation

The absolute rotation values were found to change over time following an exponential regression model, almost identical for all the different test conditions (Table 3). In the baseline condition the rotation had a time constant of 0.041°/s (*p* < 0.001). Dorsal vibration induced somewhat slower rotation than baseline with a time constant of 0.036°/s (right and left vibration, *p* < 0.001). Ventral left-sided vibration induced somewhat faster longitudinal rotation compared to baseline, 0.043°/s (left *p* < 0.001) and ventral right-sided vibration induced somewhat slower rotation compared to baseline, 0.039°/s (right *p* < 0.001). At further review, a rotation change of about 0.5°/s was observed for the first 10 s, thereafter this displacement increased to 1°/s during baseline, and during dorsal vibration and ventral left vibration, whereas ventral right vibration produced a 1°/s rotation already from the start of vibration (Fig. 2b).

The signed rotation values were found to change over time following linear regression model. A systematic rotation was not found for the baseline condition (*p* = 0.330), but the subjects rotated often equally and with the same velocity in both left and right direction. Similarly, during vibration on right side muscles, there were no systematic preference for the direction of rotation (dorsal, *p* = 0.317, ventral, *p* = 0.496). However, for left-sided vibration there was a systematic side preference for rotation direction to the right (dorsal, time constant 0.51°/s, *p* = 0.002 and ventral, time constant 0.74°/s, *p* < 0.001) (Table 3; Fig. 2c).

## Discussion

Spatial orientation has in this study been confirmed to be a highly adaptable system with regards to the interpretation of available sensory information. We were able to determine a causal relationship between a disturbed information from cervical muscles and an altered spatial orientation with muscular-specific displacement during the stepping test.

Forward displacement was the normal behavior during STIP in the absence of visual information. This displacement increased when vibration was applied on the dorsal muscles and decreased during ventral vibration. Vibration on the ventral muscles caused a faster longitudinal rotation than dorsal vibration. The vibratory perturbation thus caused illusions of movements, shown by muscular-specific displacement during STIP. The dynamic component, by means of the duration effect during different conditions, showed both altered moved distances and larger values for longitudinal rotation in the vibration conditions relative to the baseline.

### Moved distance

The dynamic component of STIP was most obvious for moved distance, both with and without vibratory perturbation. Regression analyses revealed a linear, constant forward movement for the moved distance. Notably, proprioceptive disturbances by vibration on dorsal cervical muscles increased the “natural” linear forward displacement of moved distance, whilst vibration ventrally decreased this “natural” forward displacement. The results corroborate previous studies of an increased forward displacement during dorsal cervical vibration (Ivanenko et al. 2000) with the additional finding of a slower forward displacement when vibrating ventral muscles (Table 3; Fig. 2a).

Since proprioception supply information about the head on trunk position, the effect of vibration on cervical muscles could be assumed to impact the spatial orientation according to the muscle’s function. Vibration is thought to affect the muscle spindles and simulate elongation. Vibration towards the dorsal muscles will consequently be interpreted as a dorsal movement of the torso relative to the head, and vice versa. Probably, during the STIP, the perception of this simulated muscle elongation is actively being compensated for, i.e., efforts are made to counter the simulated movement. Accordingly, our results suggest that dorsal vibration resulted in more forward displacement, whilst ventral stimulation resulted in a diminished effect.

### Longitudinal rotation

Vibration-induced-rotation appeared to be side specific (Tables 1, 2). For left side vibration there was a systematic side preference to the right (Table 3; Fig. 2b). Moreover, in absolute values the rotational effect was obvious during all test conditions (Table 3; Fig. 2c). However, vibration on the ventral muscles resulted in faster rotation than dorsal vibration (Table 3). The vibratory stimulation seemed to induce the rotational displacement in an exponential fashion, i.e., for each of the first 10 s with a time constant of about 0.5°/s and thereafter with 1°/s (Fig. 2c) in corroboration with findings by Bove and coworkers (Bove et al. 2002). Hence, these findings indicate that the ability to sense rotation after a certain point of time might dramatically be reduced. Thus, the perception of current position was maintained during the initial period during STIP to gradually deteriorate over time, especially during vibration on the ventral sternocleidomastoid muscle—a powerful rotator of the neck. The late onset of the vibratory effect could possibly be explained by retained spatial information for the first 10 s, after which the proprioceptive sensory illusions became too powerful. It could be speculated that the rotational displacement occurred due to an increased reliance on proprioceptive information and a less reliance on vestibular information in the actual setting with no visual and auditory cues.

Recently, side preferences has also been reported by Paquet and coworkers during rotation, linked to preferred foot in the Fukudas stepping test (Paquet et al. 2017). Our subjects were all right-handed, and handedness can possibly be linked to preferred leg/foot. We could, therefore, assume that side predominance could underlie a side preference for our group as well. We have earlier found some asymmetry in movement detection in head on trunk repositioning, favoring a left side dominance (Malmstrom et al. 2009). For the non-vibrated condition, direction of rotation was found to be leftwards (Fig. 2b), a side preference also described by Bonanni (1998).

However, during vibration we found a systematic muscle specific influence and we, therefore, postulate that the findings during these conditions were related to the actual proprioceptive perturbation. In summary, we found larger rotation when the left side was stimulated, and a larger moved distance when the right side was stimulated. Whether these side differences have anything to do with hemispherical dominance would be interesting to elucidate in the future.

When the direction of rotation was normalized into absolute values, the effect of vibration was more consistent (Fig. 2c). Supported by functional anatomy, one would expect perturbations to the ventral sternocleidomastoid muscle to alter spatial orientation along the longitudinal axis more than dorsal paraspinal perturbations, and so was the case (Fig. 2c).

The cervical proprioceptive influence on spatial displacement has earlier been investigated during stepping and walking by Bove and coworkers (Bove et al. 2001, 2002). They found muscle specific displacement, explained by modification of the body-centered coordinates in an internal model. Our results favor a muscle specific displacement, but not from a “spatial neutral position”, but rather with individual variations in the baseline condition (Table 3; Fig. 2). Natural individual variations have earlier been reported between subjects (Paquet et al. 2017).

## Method

We controlled that no external cues during the STIP assisted spatial orientation perception; the instructions came throughout the test from the same distance and directly from front, the helmet (150 g) was mounted centered, all vibrators were attached during the whole test and cables were lifted during the test not to tighten (Fig. 1 a).

Except for the baseline condition, where the first stepping test excelled, there was no significant repetition effect within the rest of the test conditions. The significant repetition effect in the baseline condition disappeared when STIP I was omitted. For the vibration conditions with each three STIP performances, no repetition effect was found.

The distance of displacement during moved distance could possibly be shorter if the subjects held their arms in 90° elevation as Fukuda (1959) and Bonanni (1998). However, we adopted arms alongside in accordance with Paquet (2014) since we wanted to minimize the risk of fatigue in the neck and shoulder muscles which were perturbed. We also wanted to reduce the need for control of a retained arm position during performance and thereby to change focus from stepping in place. The arm position was consistently alongside the body for all STIP and would, therefore, not have an impact on the results.

We considered all subjects healthy, as stated by themselves and confirmed with an anamnestic checkup. We did not check for vestibular asymmetry, a possible confounder (Kristinsdottir et al. 2001). Our subjects; however, were at an age where vestibular asymmetry due to age-related dysfunction is less common and thus conclude the results to originate from the perturbation of cervical proprioception.

Since all STIP was performed at the same cadence, we assume the displacement, expressed as forward displacements and rotation around the longitudinal axis, to be a spatial, compensatory mechanism to correct for perceived movements, triggered by disturbances of vibration on the cervical muscles.

### Clinical implications

Postural control disturbances, investigated in the standing position, have been found in several cervical pain conditions (Karlberg et al. 1995; Giacomini et al. 2004; Stapley et al. 2006). However, postural disturbances prevail when in motion. The results from this study suggest the stepping-in-place test to be able to detect changes related to spatial orientation during motion. Since visual and auditory information was omitted and mechanoreceptive information reduced during the stepping in place, we suggest this test setup to reflect how internal reference frames are used for spatial orientation. The importance of somatosensory information for the control of postural stability, expressed in more explorative patterns during locomotion has lately been reported by Chien and coworkers (Chien et al. 2016) and favor examinations during motion as a complement to quite stance.

The first STIP (I) in the baseline condition, i.e., the first test condition in the test setup, differed from the rest of the tests. During this first STIP the subjects were probably influenced by available reference frames before the test started, i.e., having a visual (external) memory of the test area. After the first STIP the performance changed which could reflect a diminished memory (external guidance) with a shift towards more reliance on internal reference frames (Ruotolo et al. 2016). A possible shift into greater reliance on proprioception suggest a reweighting of available sensory information. This reweighting has earlier been proposed by Tjernström and coworkers, and might explain the order effect (Tjernstrom et al. 2010).

Our results support a causal relationship between disrupted cervical proprioceptive information and altered perception of spatial orientation. In its extension, this expression of disorientation might have clinical implications also when dealing with cervical pain conditions, i.e., disrupted cervical proprioception could cause imbalance and even dizziness due to sensory mismatch (Reason 1978; Brandt and Bronstein 2001). Since the results suggest that cervical proprioceptive disturbances impact spatial orientation, the present study indirectly supports disturbed cervical proprioception as a possible cause for dizziness of cervicogenic origin (Bove et al. 2001); (Wrisley et al. 2000; Brandt and Bronstein 2001). Cervical muscle fatigue, commonly present in cervical pain conditions, has also been demonstrated to effect spatial orientation during locomotion (Schmid 2005) and head on trunk orientation (Malmstrom et al. 2010).

The actual findings support the importance of proprioception on dynamic properties of egocentric/internal spatial reference frames during locomotion and also favor sensory reweighting in spatial orientation. The findings might increase the understanding about how cervical pain and disability interact with imbalance and dizziness. Since disturbed proprioception impair spatial orientation it can consequently affect the postural control system. As cervical muscle disturbances affects spatial orientation it could, therefore, cause imbalance or dizziness due to the sensory mismatch theory (Reason 1978).

## Conclusion

The results reveal a causal relationship between cervical proprioception and spatial orientation, supported by muscle specific displacement during perturbation. The moved distance was significantly larger when the vibration was applied on the dorsal paraspinal muscles relative to on the ventral sternocleidomastoid muscles and the rate of displacement was significantly larger during dorsal paraspinal muscle vibration. Rotation to the right was induced during left-sided vibration and the rate of rotation was significantly larger for ventral than dorsal vibration. Hence, disturbed cervical proprioception affects the dynamic properties of spatial orientation and initiate reweighting based on available sensory information.

## Abbreviation

STIP: Stepping-in-place
